# Blood chemokine profile in untreated early rheumatoid arthritis: CXCL10 as a disease activity marker

**DOI:** 10.1186/s13075-017-1224-1

**Published:** 2017-02-02

**Authors:** Jayesh M. Pandya, Anna-Carin Lundell, Kerstin Andersson, Inger Nordström, Elke Theander, Anna Rudin

**Affiliations:** 1 Department of Rheumatology and Inflammation Research, Institute of Medicine, Sahlgrenska Academy of University of Gothenburg, Box 480, S-405 30 Gothenburg, Sweden; 2 Department of Rheumatology, Skåne University Hospital Lund and Malmö, Lund University, Lund, Sweden

**Keywords:** Chemokine, Chemokine receptor, CXCL10, T cell, Rheumatoid arthritis, Multivariate discriminant analysis

## Abstract

**Background:**

We have recently analyzed the profile of T-cell subtypes based on chemokine receptor expression in blood from untreated early rheumatoid arthritis (ueRA) patients compared to healthy controls (HC). Here, we compared the levels of the respective chemokines in blood plasma of ueRA patients with those of HC. We also studied the association of chemokine levels with the proportions of circulating T-cell subsets and the clinical disease activity.

**Methods:**

Peripheral blood was obtained from 43 patients with ueRA satisfying the ACR 2010 criteria and who had not received any disease-modifying anti-rheumatic drugs (DMARD) or prednisolone, and from 14 sex- and age-matched HC. Proportions of T helper cells in blood, including Th0, Th1, Th2, Th17, Th1Th17, TFh, and regulatory T cells, were defined by flow cytometry. Fifteen chemokines, including several CXCL and CCL chemokines related to the T-cell subtypes as well as to other major immune cells, were measured in blood plasma using flow cytometry bead-based immunoassay or ELISA. Clinical disease activity in patients was evaluated by assessing the following parameters: Disease Activity Score in 28 joints (DAS28), Clinical Disease Activity Index (CDAI), swollen joint counts (SJC), tender joint counts (TJC), C-reactive protein (CRP), and erythrocyte sedimentation rate (ESR). The data were analyzed using multivariate factor analyses followed by univariate analyses.

**Results:**

Multivariate discriminant analysis showed that patients with ueRA were separated from HC based on the blood plasma chemokine profile. The best discriminators were CXCL9, CXCL10, CXCL13, CCL4, and CCL22, which were significantly higher in ueRA compared to HC in univariate analyses. Among the chemokines analyzed, only CXCL10 correlated with multiple disease activity measures, including DAS28-CRP, DAS28-ESR, CDAI, SJC in 66 joints, CRP, and ESR.

**Conclusions:**

High circulating levels of CXCL10 in the plasma of ueRA patients and the association with the clinical disease activity suggests that CXCL10 may serve as a disease activity marker in early rheumatoid arthritis.

**Electronic supplementary material:**

The online version of this article (doi:10.1186/s13075-017-1224-1) contains supplementary material, which is available to authorized users.

## Background

Rheumatoid arthritis (RA) is a chronic and systemic inflammatory disease characterized by synovial inflammation and progressive destruction of joint cartilage and bones [1, 2]. The etiopathogenesis of RA is not fully understood, but various leukocytes including T cells, B cells, monocytes, neutrophils, eosinophils, dendritic cells (DC), and natural killer (NK) cells, as well as soluble factors such as cytokines and chemokines, are suggested to be involved in disease pathogenesis [2–4]. It is believed that early RA constitutes a ‘window of opportunity’ when appropriate therapy may halt the irreversible damage and prevent the switch from acute resolving inflammation to chronic and persistent inflammation [5, 6]. However, our current understanding of pathogenic mechanisms in early and preclinical RA is even less than that in established RA.

In the current paradigm of the T-cell subsets, T helper (Th)1 and Th17 cells are mainly described as contributing to RA pathogenesis [2, 3, 7]. However, it has been shown that the profile of synovial fluid (SF) cytokines in very early RA is different from that in established RA, and has a Th2 and Th17 bias (i.e., displays elevated concentration of interleukin (IL)-2, IL-4, IL-13, IL-17, IL-15, basic fibroblast growth factor, and epidermal growth factor compared to established RA) [8]. It has also been shown that untreated patients with very early RA (disease duration less than 6 weeks) display increased serum levels of cytokines related to Th17 polarization and neutrophil recruitment and activation compared to healthy controls (HC) [9]. Another study using T-cell clones from synovial fluid found that IL-4 production was predominant in early RA patients, whereas interferon (IFN)γ production was predominant in established RA patients [10]. These findings point to differences in the profile of inflammatory mediators in early RA versus established RA. Recently, we have performed a broad-scale analysis of blood T-cell subtypes based on chemokine receptor expression in patients with untreated early rheumatoid arthritis (ueRA) compared to HC [11]. The analyzed T-cell subsets included well-defined Th1, Th2, Th17, (T follicular helper (TFh), Th1Th17 and regulatory T cell (Treg) subsets, and also unconventional T-cell subsets as such CXCR3^+^Th17, CXCR3^+^Th2, and CCR4^neg^CXCR3^neg^CCR6^+^ (denoted CCR6^+^ only). The above study from our group demonstrated that the balance of T-helper subsets in the circulation of ueRA patients is skewed towards Th2 and Th17 cells.

During synovial inflammation, leukocyte trafficking to synovial tissue is a key pathogenic process in RA, which is mediated by chemokines and chemokine receptors [3, 12]. Published studies on the chemokine levels in the circulation or in the synovium have not sub-classified patients into early versus established RA, or treated versus untreated groups. For example, a study including patients with early RA investigated chemokines in serum, but 41% of the patients were treated with disease-modifying anti-rheumatic drugs (DMARD) [13]. Similarly, in another cohort with pre-patients and early RA patients in which blood chemokines were investigated, 39% of the patients were treated with prednisolone [14]. Treatment with biological and non-biological DMARD in RA have been shown to alter the chemokine levels in the blood [15–18], suggesting a need to evaluate chemokines in a pure group of DMARD-naive ueRA patients.

In our previous study in ueRA patients, T-helper cell subsets could be defined based on a combination of four chemokine receptors, i.e., CCR4, CCR6, CXCR3, and CXCR5 in the CD4^+^ or CD4^+^CD45RA^neg^ compartment [11]. In this study we therefore questioned if any of the chemokines that bind to the above chemokine receptors are differentially present in the blood of ueRA patients compared to HCs and how they relate to T-cell subsets and clinical disease activity. We analyzed the following 15 chemokines (cells expressing corresponding chemokine receptor shown in brackets): CXCL9, CXCL10, and CXCL11 (Th1 and NK cells), CCL2, CCL3, CCL4, and CCL5 (Th2, macrophages, NK cells), CCL17 and CCL22 (Th2 and Tregs), CCL20 (Th17, B cells, and dendritic cells), CXCL13 (TFh and B cells), CCL11 (eosinophils and basophils), and CXCL1, CXCL5, and CXCL8 (neutrophils). We also investigated how chemokine levels were related to T-cell subset proportions and clinical disease activity in ueRA. By the use of multivariate analysis, we found that the ueRA patients were separated from HCs based on their chemokine profile. We also found that the circulating levels of CXCL9, CXCL10, CXCL13, CCL4, and CCL22 were significantly higher in ueRA compared to HCs. Among the discriminator chemokines, only CXCL10 correlated with clinical disease activity.

## Methods

### Patients and healthy controls

The patient group comprised of 43 subjects who were newly diagnosed with RA according to the ACR/EULAR 2010 criteria and who were all untreated (ueRA). The inclusion criteria were as follows: ≥18 years old, ≥2 swollen joints (SJC 66) and ≥2 tender joints (TJC 68) (based on 66/68 joint count, respectively), rheumatoid factor (RF)-positive or anti-citrullinated protein/peptide antibody (ACPA)-positive or C-reactive protein (CRP) ≥10 mg/L, at least moderate disease activity (>3.2) by composite index Disease Activity Score in 28 joints (DAS28-CRP), symptom duration <24 months (retrospective patient-reported symptoms), and no treatment with any DMARD or prednisolone. Blood samples were drawn from the DMARD- and prednisolone-naïve patients within 1–2 weeks after RA diagnosis. The patient group was compared to a group of 14 age- and sex-matched healthy control subjects (HC). Characteristics of patients and HC are shown in Table 1. The median age of the patient cohort (61 years, range 22–78 years) and HC (62.5 years, range 35–72 years) was not significantly different (*P* = 0.68). The patients were recruited at the Rheumatology clinics at Sahlgrenska University Hospital and at Skåne University Hospital, Sweden. The study was approved by the regional ethics committee of Gothenburg, Sweden, and all patients signed an informed consent form.

**Table 1:** Baseline characteristics of early diagnosed untreated rheumatoid arthritis patients and healthy controls

	ueRA	Healthy controls
Number	43	14
Female (*n* (%))	30 (69.7)	9 (64.2)
Age (years) ^*a,b,c*^	61 (22–78)	62.5 (35–72)
Symptom duration (months) ^*a,b,d*^	6 (2–23)	ND
CRP (mg/L) ^*a,b*^	9.1 (0.3–152)	ND
ESR (mm/hour) ^*a,b*^	26 (5–108)	ND
Swollen joint count of 66 ^*a,b*^	11 (2–30)	ND
Tender joint count of 68 ^*a,b*^	14 (3–47)	ND
Swollen joint count of 28 ^*a,b*^	9 (2–22)	ND
Tender joint count of 28 ^*a,b*^	9 (0–27)	ND
DAS28-CRP ^*a,b*^	5.1 (2.7–8)	ND
DAS28-ESR ^*a,b*^	5.3 (2.6–8.4)	ND
CDAI ^*a,b*^	28.1 (11.8–62.1)	ND
ACPA ≥20 IU/ml (*n* (%))	34 (79)	ND
RF ≥20 IU/ml (*n* (%))	33 (77)	ND
ACPA ≥20 IU/ml + RF ≥20 IU/ml (*n* (%))	30 (70)	ND
ACPA <20 IU/ml + RF <20 IU/ml (*n* (%))	5 (12)	ND

^*a*^Median and ^*b*^Range

^c^ *P* = 0.68 (statistical analysis between ueRA and healthy controls, Mann–Whitney U test)

^*d*^Retrospective patient-reported pain in the joints before RA diagnosis

*ACPA* anti-citrullinated protein/peptide antibodies, *CDAI* Clinical Disease Activity Index, *CRP* C-reactive protein, *DAS28* Disease Activity Score 28, *ESR* erythrocyte sedimentation rate, *ND* not determined, *RF* rheumatoid factor, *ueRA* untreated early rheumatoid arthritis

### Clinical evaluation

Evaluation of clinical disease activity in patients was done by assessing the following parameters: swollen joint counts of 66 joints (SJC 66), tender joint counts of 68 joints (TJC 68), swollen joint counts in 28 joints status (SJC 28), tender joint counts in 28 joints status (TJC 28), CRP, erythrocyte sedimentation rate (ESR), Disease Activity Score in 28 joints (DAS28) [19] and Clinical Disease Activity Index (CDAI) [20, 21]. ACPA positivity was determined by multiplexed anti-CCP test (BioPlex from BioRad, Hercules, USA) and RF positivity was determined by nephelometry (Beckman Coulter, Brea, USA). Patients with ≥20 IU/ml anti-CCP antibodies or RF in serum were considered ACPA- or RF-positive, respectively.

### Chemokine analysis

Chemokine analysis was performed in blood plasma of ueRA patients and HCs. The chemokines CCL2 (MCP-1), CCL3 (MIP-1α), CCL4 (MIP-1β), CCL5 (RANTES), CCL17 (TARC), CCL20 (MIP-3α), CXCL1 (GROα), CXCL5 (ENA-78), CXCL8 (IL-8), CXCL9 (MIG), CXCL10 (IP-10), and CXCL11 (I-TAC) were analyzed using flow cytometry bead-based immunoassay (LEGENDplex™ Human Proinflammatory Chemokine Panel, BioLegend) according to the manufacturer’s instructions, and samples were acquired on a FACSVerse equipped with FACSuite software (BD Bioscience, San Jose, USA), and analyzed with FCAP Array software (Soft Flow, Pecs, Hungary). CCL22 (MDC) and CXCL13 (BCL) were not available in the above multiplexed assay, and therefore these chemokines were analyzed using enzyme-linked immunosorbent assay (ELISA; DuoSet, R&D Systems). CCL11 (Eotaxin) was analyzed by the use of ELISA (DuoSet) since the values obtained in the bead-based immunoassay above were too high compared to previously published levels in healthy controls.

### Definition, analysis, and characterization of T-cell subsets

Peripheral blood mononuclear cells (PBMCs) were separated from whole blood (for patients sampled within 1–2 weeks after RA diagnosis) using Lymphoprep (Axis-Shield, Oslo, Norway). For cell counts (true count; TC), we used the BD TruCOUNT Absolute Counting Tubes with the addition of CD45 PerCP and CD4 APC-H7 antibodies (BD Biosciences). T-cell subsets were defined and analyzed using flow cytometry, and the phenotypes of defined T-cell subsets were confirmed by lineage-specifying transcription factor expression by quantitative polymerase chain reaction (qPCR) and cytokine secretion by Cytometric Bead Array as described in a previous study from our group [11]. In brief, the cell surfaces of PBMCs were stained with fluorochrome conjugated monoclonal antibodies against the following molecules: CD4, CD45RA, CCR4, CCR6, CXCR3, CXCR5, CD127, and CD25; to evaluate FOXP3^+^ and CTLA-4^+^ cells, intracellular stainings were performed [11]. Stained samples were acquired using FACSCanto II equipped with FACS Diva software. Flow cytometry data were analyzed in FlowJo software (Tree Star, Ashland, USA). The gating strategy to define different T-cell subsets is previously described in detail [11], and also presented in Additional file 1 (Figure S1).

### Statistical analysis

**Fig. 1:**
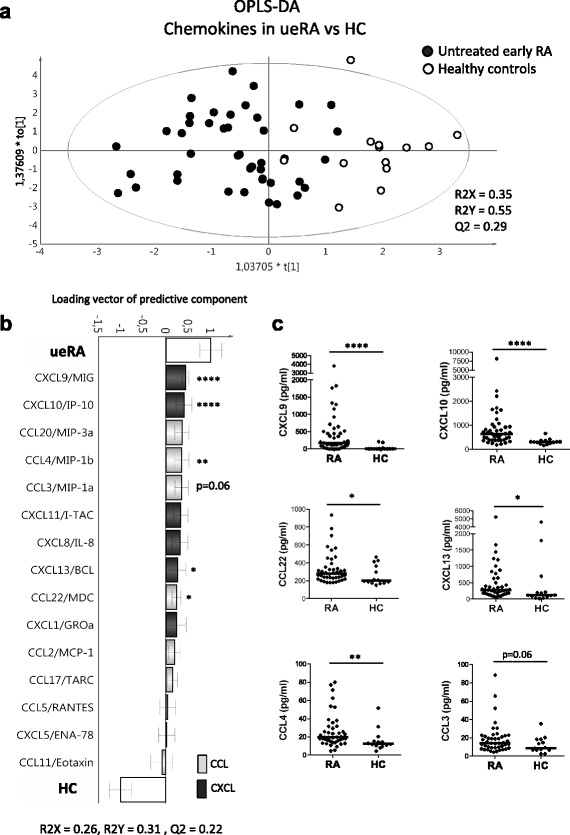
Differences in the levels of blood chemokines between untreated early rheumatoid arthritis patients and healthy controls. Multivariate factor analysis was performed to investigate differences in the blood chemokine levels between the patients with untreated early rheumatoid arthritis (*ueRA*; *n* = 43) and healthy controls (*HC*; *n* = 14). **a** Orthogonal projection to latent structures discriminant analysis (*OPLS-DA*) score scatter plot showing the separation of ueRA and HC based on differences in chemokine levels. **b** OPLS-DA column loading plot depicting the association between ueRA or HC (*y* variables) and the levels of chemokines (*x* variables). *x* variables represented by bars pointing in the same direction as ueRA are positively associated, whereas variables with bars pointing in the opposite direction are inversely related to ueRA. R2Y indicates how well the variation of *y* is explained, whereas Q2 indicates how well *y* can be predicted. **c** Univariate comparisons of the levels of CXCL9, CXCL10, CCL22, CXCL13, CCL4, and CCL3 are shown between ueRA and HC. Horizontal bars indicate median. *P ≤ 0.05, **P ≤ 0.01, ****P ≤ 0.0001, by two-tailed Mann–Whitney *U* test

**Fig. 2:**
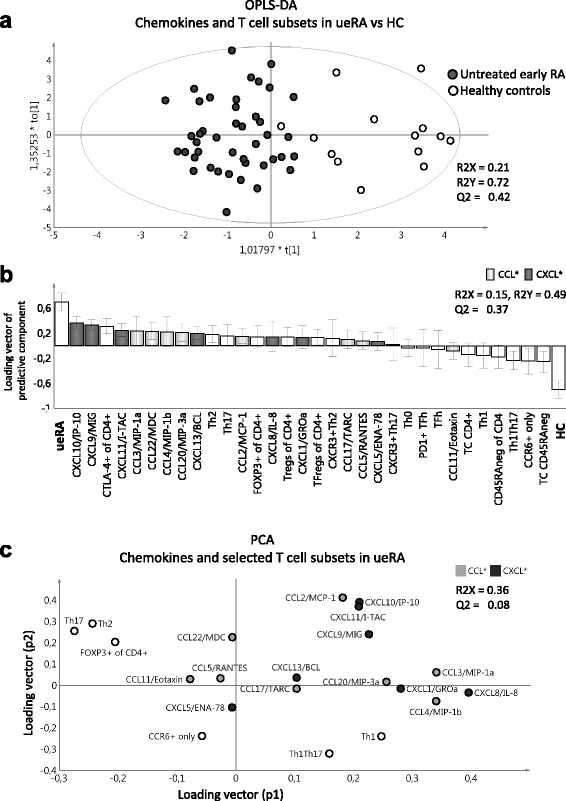
Differences in a combined blood chemokine/T-cell subset profile between untreated early rheumatoid arthritis patients and healthy controls, and the association of chemokines and T-cell subsets. Multivariate factor analysis was performed to investigate differences in combined blood chemokine levels and proportions of T-cell subset profiles between the patients with untreated early rheumatoid arthritis (*ueRA*; *n* = 43) and healthy controls (*HC*; *n* = 14). **a** Orthogonal projection to latent structures discriminant analysis (*OPLS-DA*) score scatter plot showing the separation of ueRA and HC based on differences in the combined blood chemokine/T-cell subset profile. **b** OPLS-DA column loading plot depicting the association between ueRA or HC (*y* variables) and the levels of chemokines and T-cell subset proportions (*x* variables). *x* variables represented by bars pointing in the same direction as ueRA are positively associated, whereas variables with bars pointing in the opposite direction are inversely related to ueRA. **c** Principal component analysis (*PCA*) plot of the first two principal components (p2 vs p1) depicting the association and clustering among the chemokines and T-cell subsets in the patients with ueRA. Subsets located close to each other on the same side of an axis depict positive association, while subsets located far on the opposite side of an axis or located far diagonally depict negative association

Multivariate factor analysis (SIMCA-P+ software; Umetrics, Umeå, Sweden) was used to investigate the associations of chemokines and T-cell subsets (*x* variables) with ueRA or HC, to investigate the relationship between chemokines and T-cell subsets (*x* variables) in ueRA patients, and to investigate the association between chemokines (*y* variables) and disease activity variables (*x* variables) in ueRA patients. Orthogonal projection to latent structures discriminant analysis (OPLS-DA) was implemented to examine whether ueRA patients, compared with HC, could be discriminated based on the various *x* variables (chemokines and/or T-cell subsets) examined. The two-class discrimination OPLS-DA model has one predictive component and one orthogonal component, and the default score scatter plot is t[1] vs to[1] (as shown in Figs. 1a and 2a). The scatter plot of t[1] vs to[1] is a window in the *x* space in which the separation of the two classes of observations occurs in the horizontal t[1] direction, and the vertical to[1] direction depicts the within-class variability of the separation in x-space. Principal component analysis (PCA) was performed to examine how chemokines and the proportions of T-cell subsets associate with each other in ueRA patients. OPLS was implemented to find associations between chemokines (*y* variables) and clinical disease activity measures (*x* variables) in linear multivariate models. Log transformation was used to normalize data and further scaled to unit variance in the SIMCA software by dividing each variable by 1/standard deviation (SD), so that all the variables were given equal weight regardless of their absolute value. The scale presented on the axes of the PCA and OPLS plots is a dimensionless scale, and the loading vector is normalized to length one. The quality of the OPLS models was assessed based on the parameters R2 (i.e., how well the variation of the variables is explained by the model) and Q2 (i.e., how well a variable can be predicted by the model). Univariate analyses were performed using two-tailed Mann–Whitney *U* test and two-tailed Spearman rank correlation test (GraphPad Prism Software, La Jolla, USA) as described in the figure legends. The strength of correlation was determined based on Spearman correlation coefficient (*r*) values (*r* = 0.2–0.39, weak correlation; *r* = 0.4–0.59, moderate correlation; *r* = 0.6–0.79, strong correlation; *r* >0.8, very strong correlation). A *P* value ≤ 0.05 was regarded as being statistically significant.

## Results

### Chemokine levels in blood are higher in ueRA patients than in healthy controls

First, we asked whether there were any differences in the levels of chemokines between ueRA patients and HCs. Based on the chemokines assessed, OPLS-DA demonstrated a separation between ueRA patients and HCs (Fig. 1a). The majority of patients with ueRA appeared in the two left quadrants, whereas all HCs appeared in the two right quadrants. The chemokines that displayed the strongest association (positive or negative) with ueRA patients are identified in the OPLS-DA column plot in Fig. 1b. ueRA was positively associated with higher levels of most of the chemokines analyzed, except for eotaxin, CXCL5, and CCL5 (Fig. 1b). The multivariate associations were corroborated by univariate analyses, which demonstrated that the CXCL9, CXCL10, CCL22, CXCL13, and CCL4 levels were significantly higher in ueRA compared to HCs (Fig. 1c). These results show that patients with ueRA display higher levels of several blood chemokines compared to HCs. In particular, levels of chemokines related to Th1 (CXCL9, CXCL10), Th2 (CCL22), T follicular helper and B cells (CXCL13), and macrophages (CCL4) were higher in ueRA compared to HCs.

### A combined chemokine/T-cell subset profile increases the separation between ueRA and HCs compared to the chemokine profile or T-cell subset profile alone

We have previously demonstrated that the proportion of various T-helper and T regulatory cell subsets in blood can be used to separate ueRA from HCs [11]. Here, we investigated how chemokines and T-cell subset proportions together affect the separation between ueRA and HCs. When compared to the chemokine profile alone (Q2 = 0.29) (Fig. 1a) or the T-cell subset profile alone (Q2 = 0.34; Additional file 1: Figure S2), the combined chemokine and T-cell subset profile led to a stronger separation between ueRA patients and HCs as demonstrated by the increased predictability of the latter OPLS-DA model (Q2 = 0.42) (Fig. 2a).

The OPLS-DA column plot in Fig. 2b displays the association (positive or negative) of chemokines and T-cell subsets with ueRA patients or HCs. As clearly demonstrated, most of the chemokines were elevated among the ueRA patients along with increased proportions of CTLA-4^+^CD4^+^ T cells and Th2 cells. On the contrary, none of the chemokines were higher among HC, while the absolute number of CD4^+^CD45RA^neg^ T cells (memory/mature T cells) per ml of blood, as well as the proportions of CCR6^+^ only and Th1Th17 subsets, were higher in the HC group. In the combined OPLS-DA analysis, CXCL10 among chemokines and Th2 cells among T helper subsets in blood were most strongly associated with ueRA (Fig. 2b).

In order to evaluate how chemokines and the corresponding T-cell subsets associate with each other in ueRA, a PCA analysis was performed including chemokines and selected T-cell subsets based on results from Fig. 2b. As shown in Fig. 2c, none of the chemokines clustered together with the corresponding T-cell subset. Instead, most chemokines were projected far from the relevant T-cell subsets. For example, CXCL9, CXCL10, and CXCL11 were projected on the opposite side of the *x* axis from Th1 and Th1Th17 cells. Also, CCL20 was located on the opposite side of the axis or diagonally from Th17, CCR6^+^ only, and Th1Th17 subsets. Finally, CCL2, CCL3, and CCL4 were located on the opposite side of the axis or diagonally from Th2 cells. However, no statistically significant inverse correlations were observed between chemokine levels and the corresponding T-cell subset proportions.

Overall, these results demonstrate that a combined profile of chemokines and T-cell subsets led to stronger separation between ueRA and HC compared to chemokine or T-cell subset profiles alone. However, although the levels of some chemokines and the proportions of the corresponding T-cell subsets were elevated among ueRA patients, they were not positively associated. Instead, they displayed a negative association pattern in multivariate PCA analysis.

### Only the levels of CXCL10 correlate with clinical disease activity

**Fig. 3:**
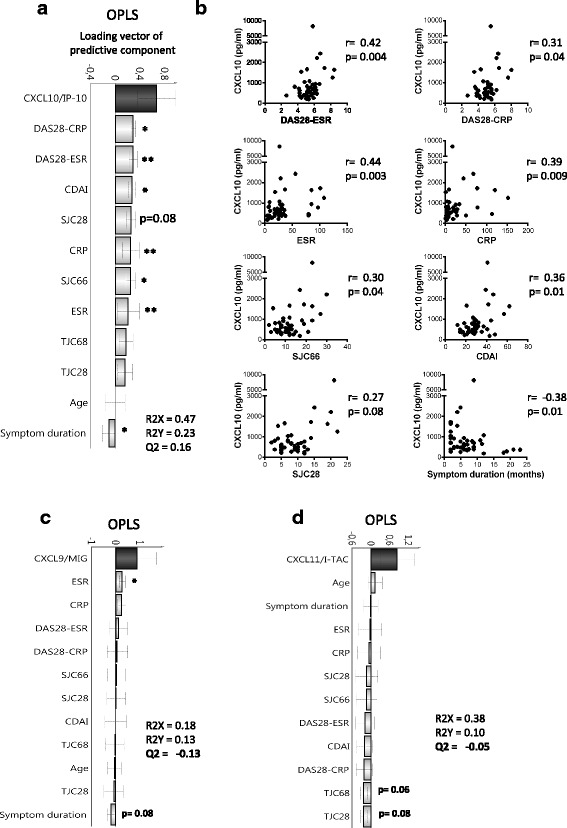
Association of Th1 chemokines with clinical disease activity in ueRA. Orthogonal projection to latent structures (*OPLS*) column loading plot depicting the association between CXCL10 (**a**), CXCL9 (**c**) or CXCL11 (**d**) (*y* variables) and clinical disease activity measures, age, and symptom duration (*x* variables) in the patients with ueRA (*n* = 43). *x* variables represented by bars pointing in the same direction as the chemokine are positively associated, whereas variables with bars pointing in the opposite direction are inversely related to the respective chemokine. A negative Q2 value in (**c**) and (**d**) indicate that these OPLS models are not predictive. **b** The correlations of CXCL10 with clinical disease activity measures in ueRA patients (Spearman rank correlation test; *r*). *P ≤ 0.05, **P ≤ 0.01. *CDAI* Clinical Disease Activity Index, *CRP* C-reactive protein, *DAS28* Disease Activity Score in 28 joints, *ESR* erythrocyte sedimentation rate, *SJC* swollen joint counts, *TJC* tender joint counts

We next investigated whether the chemokine levels in plasma were associated with clinical disease activity in ueRA patients. OPLS analysis showed that, among the chemokines that were positively associated with ueRA (Fig. 1b), CXCL10 resulted in the strongest predictive OPLS model with clinical measures (Fig. 3a). In fact, high levels of CXCL10 were found to be positively associated with all clinical disease activity measures (Fig. 3a). In univariate analyses, CXCL10 levels significantly correlated with DAS28-ESR, DAS28-CRP, CDAI, SJC 66, CRP, and ESR (Fig. 3b), and a trend towards positive correlation was seen with SJC28 (*r* = 0.27, *P* = 0.08). The levels of CXCL10 associated negatively with symptom duration in multivariate analysis (Fig. 3a), which was corroborated by univariate correlation analysis (Fig. 3b).

Although CXCL9 and CXCL11, like CXCL10, are ligands to CXCR3, CXCL9 levels were unrelated to clinical disease activity (except for ESR) (Fig. 3c) and the levels of CXCL11 displayed a trend towards negative correlation with tender joint counts (Fig. 3d). Additionally, levels of the Th17-related chemokine CCL20 displayed a positive association pattern to clinical disease activity (Additional file 1: Figure S5), while levels of the eosinophil-related chemokine eotaxin were negatively related to clinical disease activity, although none of these associations were significant in univariate correlation analyses (Additional file 1: Figure S4).

Next, we evaluated sex-based differences in the levels of chemokines in HC and ueRA patients. Although some of the chemokines in HC displayed a pattern of positive association with female sex in multivariate analysis, no significant difference in univariate analyses was found for chemokine levels between females and males in HC (Additional file 1: Figure S3). In ueRA patients, the levels of eotaxin associated negatively with female sex in multivariate analysis and female patients had significantly lower levels of eotaxin compared to male patients (Additional file 1: Figure S3) and HC females (*P* = 0.02). No significant difference was found for the levels of other chemokines between female and male patients. Sex-based differences were also observed for the association of eotaxin levels with the clinical disease activity. In female patients, eotaxin displayed a negative association with clinical disease measures in multivariate analysis and correlated negatively with DAS28-ESR, SJC66, CRP, and ESR (Additional file 1: Figure S4). In contrast, in male patients eotaxin displayed a pattern of positive association with clinical disease measures in multivariate analysis; however, no significant correlation could be seen in univariate analyses (Additional file 1: Figure S4). Furthermore, CCL20 correlated positively with DAS28-ESR, CRP, and ESR in female patients but not in male patients (Additional file 1: Figure S5). Sex-based differences were not observed for other chemokines and their associations with clinical disease activity (data not shown).

We also examined whether there were any differences in the chemokine levels between ACPA^+^ and ACPA^neg^ ueRA patients, or between RF^+^ and RF^neg^ ueRA patients, or between seropositive (ACPA^+^ and/or RF^+^) ueRA and seronegative (ACPA^neg^ and RF^neg^) ueRA patients. However, we did not find any predictive OPLS-DA model in which chemokine levels could be associated with the above-stated ueRA subgroups (Additional file 1: Figure S6). In univariate comparisons, only the levels of CCL4 were significantly higher in ACPA^+^ ueRA patients compared to ACPA^neg^ ueRA patients, and only the levels of CCL17 were significantly higher in RF^+^ ueRA patients compared to RF^neg^ ueRA patients. The levels of other chemokines did not differ between the above ueRA subgroups. Thus, the presence of RF or ACPA in the serum did not seem to interfere with the measurement of the chemokines in our study.

**Fig. 4:**
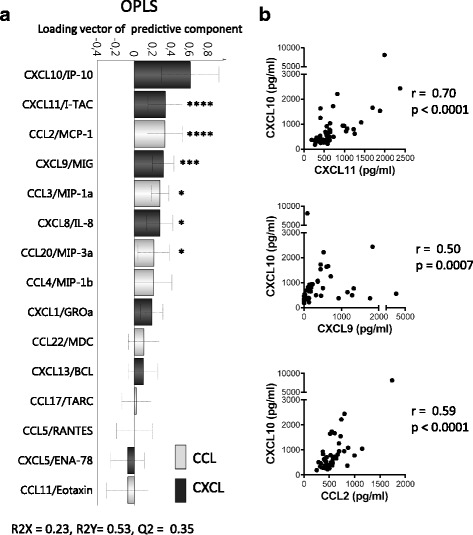
Association of CXCL10 with other chemokines in untreated early rheumatoid arthritis patients. **a** Orthogonal projection to latent structures (*OPLS*) column loading plot depicting the association between the levels of CXCL10 (*y* variables) and other chemokines (*x* variables) in patients with ueRA (*n* = 43). *x* variables represented by bars pointing in the same direction as CXCL10 are positively associated, whereas variables with bars pointing in the opposite direction are inversely related to CXCL10. **b** Correlation analyses between the levels of CXCL10 and other chemokines in ueRA patients (Spearman rank correlation test; *r*). *P ≤ 0.05, *** *P* ≤ 0.001, **** *P* ≤ 0.0001

Since CXCL10 correlated with clinical disease activity (Fig. 3a and b), OPLS and correlation analyses were performed to investigate the association of CXCL10 with other chemokines (Fig. 4). CXCL10 displayed a strong correlation with CXCL11 and moderate correlations both with CCL2 and CXCL9 (Fig. 4). CXCL10 also displayed weak correlations with CCL3 (*r* = 0.35, *P* = 0.02), CXCL8 (*r* = 0.32, *P* = 0.03), and CCL20 (*r* = 0.33, *P* = 0.02) (Fig. 4). Taken together, although the levels of CXCL10 (a Th1-related chemokine) correlated with several of the other chemokines assessed, only the levels of CXCL10 correlated with multiple clinical disease activity measures in ueRA patients.

## Discussion

Chemokines and chemokine receptors are involved in leukocyte migration and inflammatory processes, and are therefore suggested to be involved in RA pathogenesis. Indeed, in this study we found that the patients with ueRA were separated from HC in multivariate discriminant analysis based on the blood chemokine profile. A combined profile of chemokines and T-cell subsets led to stronger separation between ueRA and HC compared to chemokine or T-cell subset profiles alone. The levels of chemokines related to Th1 (CXCL9, CXCL10), Th2 (CCL22), T follicular helper and B cells (CXCL13), and macrophages (CCL4) were significantly higher in ueRA compared to HC, pointing to the role of these chemokines and immune cells in early RA pathogenesis. Among the discriminator chemokines, only the levels of CXCL10 correlated with several clinical disease activity parameters, including DAS28-CRP, DAS28-ESR, CDAI, SJC in 66 joints, CRP, and ESR.

In line with our results, previous studies have reported higher levels of CXCL10 in serum or plasma in patients with both established RA [16] and early RA [13, 14] compared to healthy controls. However, a significant proportion of these patients were treated with DMARD when chemokine levels were assessed. Similarly, higher serum levels of CCL22 in patients with RA compared to healthy controls have been reported previously; however, in that study, the majority of the patients were treated with DMARD and there was no information concerning the disease duration [22]. Various treatments have been shown to affect the levels of chemokines in the circulation [15–18], which suggests that evaluation of chemokines in untreated patients might be advantageous.

Among the evaluated chemokines, only the plasma levels of CXCL10 correlated with the clinical disease activity in our cohort. In a previous study in established RA, a correlation between CXCL10 and DAS28-CRP has been seen, but no correlation with other clinical disease activity measures such as swollen joint counts, CRP, and ESR was observed [15]. Another study in established RA did not observe any correlation between CXCL10 and clinical disease activity measures [18]. Thus, ours is the first study that defines CXCL10 as a disease activity marker in early RA by demonstrating correlation between CXCL10 and multiple clinical disease activity measures. The reduction in CXCL10 with symptom duration and correlations with multiple clinical disease activity measures in early RA but not in established RA suggests that CXCL10 plays a more critical role in the early stages of the disease, and can function as a disease activity marker in early RA.

CXCL10 (traditionally known as IFNγ-inducible 10-kd protein or IP-10) can be secreted by several cell types such as endothelial cells, fibroblasts, monocytes, neutrophils, dendritic cells, mesenchymal cells, keratinocytes, astrocytes, and so forth [23–25]. CXCL10 can be induced in response to IFNγ, IFN-α/β, IL-1β or tumor necrosis factor (TNF) [26, 27]. Interestingly, the type I interferon (IFN) signature has been demonstrated as a biomarker of preclinical RA [28], and granulocytes are shown to be a major contributor of this signature in early arthritis [29]. Since IFN-α is a potent inducer of CXCL10 [26], high levels of CXCL10 in early RA, despite lower proportions of Th1 cells, may suggest that type I IFN-dependent immune mechanisms could be important in preclinical and early RA. Moreover, the role of Th17 cytokines is well established in neutrophil activation and, according to recent models, Th2 and Th17 pathways may amplify each other in certain conditions [30]. Thus, the dominance of Th2 and Th17 milieu may induce a granulocyte-mediated type I IFN response, which may further induce downstream secretion of CXCL10 and other chemokines contributing to early RA pathogenesis.

CXCL10 can potentially regulate inflammation at several levels, contributing to RA pathogenesis and disease activity. CXCL10 and CXCR3 play crucial roles in leukocyte homing to inflamed tissue and also in perpetuation of inflammation and tissue damage [25]. In particular, CXCL10 promotes directional migration of activated T cells, monocytes, and NK cells, induces integrin activation, and promotes T-cell adhesion to endothelial cells [31, 32]. It can therefore coordinate recruitment of various immune cells to the site of inflammation. CXCL10 can also induce RANKL expression in RA synoviocytes and CD4^+^ T cells [33], which may induce bone resorption. It has been shown that stimulation of fibroblast-like synoviocytes (FLS) with CXCL10, CXCL9, and CCL2 enhances the proliferation of these cells, which may lead to synovial hyperplasia [34]. Chemokines such as CXCL10, CXCL9, CCL5, CCL4, and CCL2 can also stimulate FLS and chondrocytes to release inflammatory mediators including cytokines, matrix metalloproteinases (MMPs), and other enzymes, leading to degradation of the extracellular matrix and cartilage [34–36]. Higher levels of CXCL10 and other chemokines have indeed been detected both in synovial fluid and synovial tissue of RA patients compared to osteoarthritis patients [37, 38]. In patients with established RA, a chemokine gradient with higher levels in synovial fluid compared to blood has been reported for CXCL10, CXCL9, CCL2, CCL3, CCL4, and CXCL8 [38]. Among these, differences in the levels of chemokines were most dramatic for CXCL10 and CXCL8 (>10 times higher concentration in RF synovial fluid compared to RA serum for both chemokines) [38]. The relative levels of chemokines between synovial and peripheral blood compartments in early RA and how a chemokine gradient affects the selective recruitment of immune cell subtypes in the joints of patients with early RA has not been established.

We here show that the levels of CXCL10 levels correlated strongly with those of CXCL11 and moderately with CCL2 and CXCL9. However, only CXCL10 levels correlated with clinical disease. This suggests that the inflammation and disease severity in patients are directly associated with the levels of CXCL10 but not with the other CXCR3 ligands or chemokines. Supporting this notion, a recent genetic association study based on single nucleotide polymorphisms (SNPs) showed that the CXCL10 GG genotype was an independent factor associated with increased probability of extra-articular manifestation development, while CXCL9 genotypes did not display such an association [39]. In a study with synovial fibroblast cell lines from RA patients, significant CXCL9 and CXCL10 secretion, but not CXCL11, could be observed upon IFNγ stimulation, and only CXCL10 secretion, but not CXCL9 and CXCL11, was found upon TNF or IL-1β stimulation [27]. However, when these cells were stimulated with a combination of IFNγ and TNF, significant secretion of all three chemokines (CXCR3 ligands) was observed [27]. Thus, despite differential regulation of CXCL10 and other chemokines in chronic inflammation, association among several chemokines can be seen due to synovial hyperplasia and paracrine activity/positive feedback loop among various cytokines and chemokines.

It has been reported previously that the serum levels of CXCL13 can act as a disease activity marker in early RA [40, 41]. However, we did not observe any significant correlations between CXCL13 and clinical disease activity measures in our cohort. The reasons for these different results are unclear, but could be due to differences in the percentages of ACPA^+^ and RF^+^ patients which varied between our cohort and patient cohorts in previous studies. The patient cohort in the present study had 79% ACPA^+^ and 77% RF^+^ patients, while the study by Bugatti et al. [41] had 49% ACPA^+^ and 57% RF^+^ patients, and the study by Greisen et al. [40] had 63% ACPA^+^ and 71% RF^+^ patients. However, the levels of CXCL13 were not significantly different between ACPA^+^ and ACPA^neg^ or RF^+^ and RF^neg^ subgroups in our patient cohort.

Lower levels of eotaxin were found in female patients compared to male patients and HC females in our study cohort. Interestingly, eotaxin associated negatively with clinical disease activity in female patients while it displayed a positive association pattern in male patients. High serum levels of eotaxin have been shown to associate with less radiographic progression in an early rheumatoid arthritis patient cohort which had 77.4% female patients [42]. Based on our results, it would be interesting to evaluate sex-based differences in immune cells and soluble inflammatory mediators in larger cohorts of patients.

A negative association pattern was seen between chemokines and the corresponding T-cell subsets in the present study, which could have several explanations. Chemokine-mediated apoptosis of T cells could be one reason, as it has been shown that chemokines are able to induce apoptosis in T cells depending on the co-stimulating signals and the balance of downstream signaling pathways [43, 44]. Alternatively, binding of chemokines to the chemokine receptor-expressing cells may lead to lower concentrations of chemokine protein in the blood. Another possibility is the migration of T cells to target organs such as joints, but this should depend more on the chemokine gradient between organ tissue and blood and not on the absolute plasma levels of chemokines.

Targeting of chemokines and chemokine receptors have given favorable results in preclinical studies performed in animal models of arthritis [4]. However, human clinical trials targeting CCR2, CCR5, CCL2, and CXCL8 by small molecules or monoclonal antibodies have failed to demonstrate clinical efficacy in RA (reviewed in [4]). In contrast, one publication of a phase II clinical trial using anti-CXCL10 monoclonal antibody (MDX-1100) in established RA patients who had responded inadequately to methotrexate (MTX) treatment showed that the response rate was significantly higher in MDX-1100-treated patients at week 12 according to the American College of Rheumatology 20% criteria for improvement compared to the placebo group [45].

## Conclusions

Results from our study support a critical role of CXCL10 in the inflammatory cascade. The correlation of CXCL10 with clinical disease activity and with other chemokines in ueRA patients suggests that CXCL10 plays a central role in early RA inflammation and may serve as a disease activity marker in early RA.

## Additional file

Additional file 1: Figure S1. Gating strategy describing T-cell subsets. Figure S2. OPLS-DA score scatter plot showing differences in the profile of T-cell subset proportions between untreated early RA and healthy controls. Figure S3. OPLS-DA column loading plots and univariate analyses showing sex-based differences in the levels of blood chemokines in healthy controls and untreated early RA patients. Figure S4. OPLS plots showing association of eotaxin with clinical disease activity in untreated early RA and sex-based differences. Figure S5. OPLS plots showing association of CCL20 with clinical disease activity in untreated early RA and sex-based differences. Figure S6. OPLS-DA column loading plots showing comparison in the levels of blood chemokines between untreated early RA subgroups based on ACPA or RF positivity. (PPTX 655 kb)

## Abbreviations

ACPA: Anti-citrullinated protein/peptide antibody
CDAI: Clinical Disease Activity Index
CRP: C-reactive protein
DAS28: Disease Activity Score in 28 joints
DMARD: Disease-modifying anti-rheumatic drugs
ESR: Erythrocyte sedimentation rate
HC: Healthy controls
OPLS-DA: Orthogonal projection to latent structures discriminant analysis
PBMC: Peripheral blood mononuclear cell
PCA: Principal component analysis
RA: Rheumatoid arthritis
RANKL: Receptor activator of nuclear factor kappa-B ligand
RF: Rheumatoid factor
SJC 66: Swollen joint counts of 66
TFh: T follicular helper
Th: T helper
TJC 68: Tender joint counts of 68
Treg: regulatory T cell
ueRA: Untreated early rheumatoid arthritis
