# Supplementary material for: Blood chemokine profile in untreated early rheumatoid arthritis: CXCL10 as a disease activity marker
Source: Arthritis Res Ther. 2017 Feb 2;19:20. doi: 10.1186/s13075-017-1224-1 (PMC5289001; doi:10.1186/s13075-017-1224-1)
Supplement: Additional file 1: — Figure S1. Gating strategy describing T-cell subsets. Figure S2. OPLS-DA score scatter plot showing differences in the profile of T-cell subset proportions between untreated early RA and healthy controls. Figure S3. OPLS-DA column loading plots and univariate analyses showing sex-based differences in the levels of blood chemokines in healthy controls and untreated early RA patients. Figure S4. OPLS plots showing association of eotaxin with clinical disease activity in untreated early RA and sex-based differences. Figure S5. OPLS plots showing association of CCL20 with clinical disease activity in untreated early RA and sex-based differences. Figure S6. OPLS-DA column loading plots showing comparison in the levels of blood chemokines between untreated early RA subgroups based on ACPA or RF positivity. (PPTX 655 kb) [file 13075_2017_1224_MOESM1_ESM.pptx]

## Slide 1
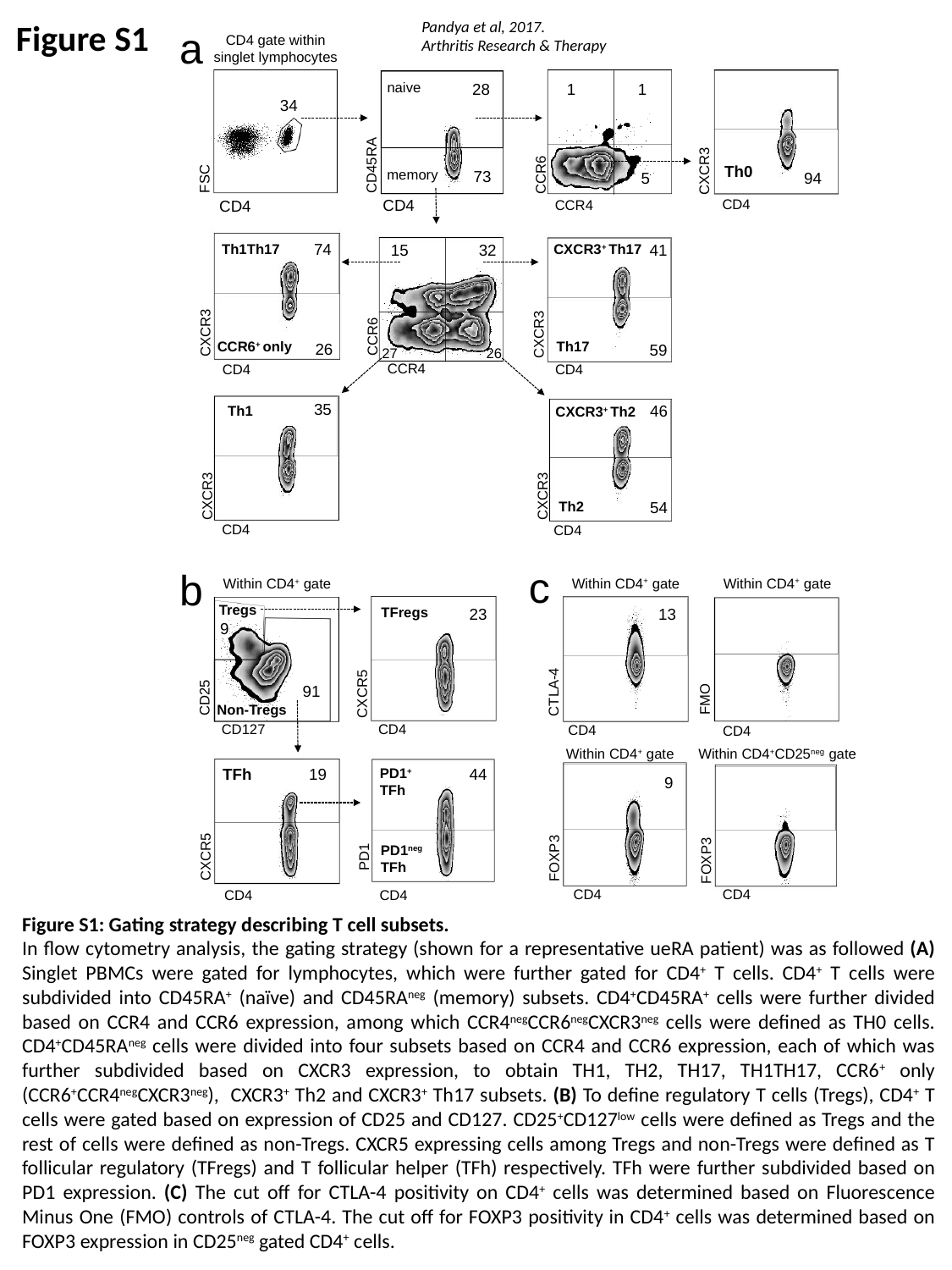

Figure S1
Pandya et al, 2017.
Arthritis Research & Therapy
a
CD4 gate within
singlet lymphocytes
naive
28
1
1
34
CD45RA
Th0
CXCR3
73
memory
CCR6
5
94
FSC
CD4
CD4
CD4
CCR4
74
Th1Th17
CXCR3+ Th17
15
32
41
CXCR3
CXCR3
CCR6
Th17
CCR6+ only
26
59
27
26
CCR4
CD4
CD4
35
46
Th1
CXCR3+ Th2
CXCR3
CXCR3
Th2
54
CD4
CD4
c
b
Within CD4+ gate
Within CD4+ gate
Within CD4+ gate
Tregs
TFregs
23
13
9
91
CTLA-4
CXCR5
CD25
 FMO
Non-Tregs
CD4
CD127
CD4
CD4
Within CD4+ gate
Within CD4+CD25neg gate
TFh
19
PD1+
TFh
44
9
PD1neg
TFh
PD1
CXCR5
FOXP3
FOXP3
CD4
CD4
CD4
CD4
Figure S1: Gating strategy describing T cell subsets.
In flow cytometry analysis, the gating strategy (shown for a representative ueRA patient) was as followed (A) Singlet PBMCs were gated for lymphocytes, which were further gated for CD4+ T cells. CD4+ T cells were subdivided into CD45RA+ (naïve) and CD45RAneg (memory) subsets. CD4+CD45RA+ cells were further divided based on CCR4 and CCR6 expression, among which CCR4negCCR6negCXCR3neg cells were defined as TH0 cells. CD4+CD45RAneg cells were divided into four subsets based on CCR4 and CCR6 expression, each of which was further subdivided based on CXCR3 expression, to obtain TH1, TH2, TH17, TH1TH17, CCR6+ only (CCR6+CCR4negCXCR3neg), CXCR3+ Th2 and CXCR3+ Th17 subsets. (B) To define regulatory T cells (Tregs), CD4+ T cells were gated based on expression of CD25 and CD127. CD25+CD127low cells were defined as Tregs and the rest of cells were defined as non-Tregs. CXCR5 expressing cells among Tregs and non-Tregs were defined as T follicular regulatory (TFregs) and T follicular helper (TFh) respectively. TFh were further subdivided based on PD1 expression. (C) The cut off for CTLA-4 positivity on CD4+ cells was determined based on Fluorescence Minus One (FMO) controls of CTLA-4. The cut off for FOXP3 positivity in CD4+ cells was determined based on FOXP3 expression in CD25neg gated CD4+ cells.

## Slide 2
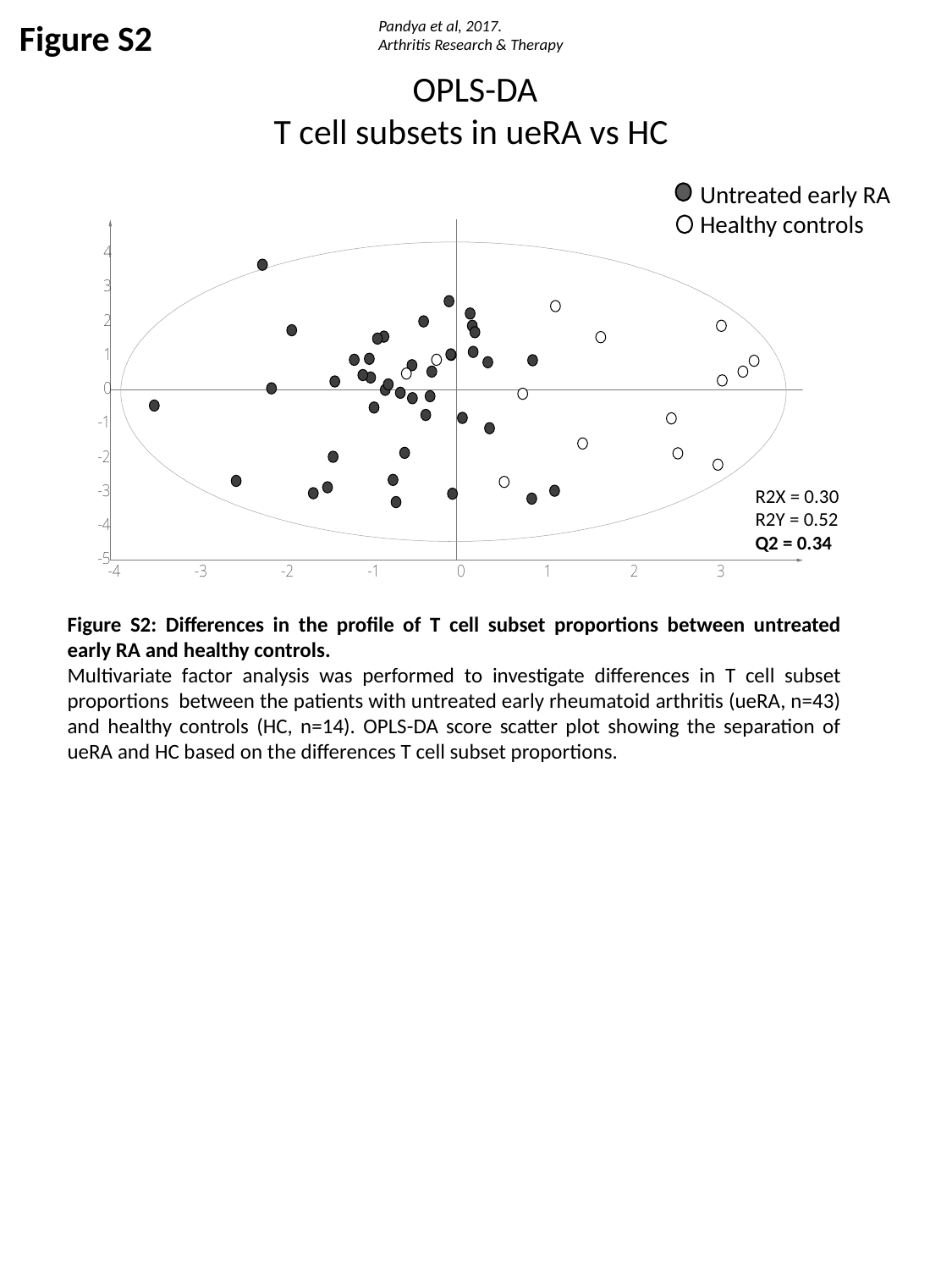

Figure S2
Pandya et al, 2017.
Arthritis Research & Therapy
 OPLS-DA
T cell subsets in ueRA vs HC
Untreated early RA
Healthy controls
R2X = 0.30
R2Y = 0.52
Q2 = 0.34
Figure S2: Differences in the profile of T cell subset proportions between untreated early RA and healthy controls.
Multivariate factor analysis was performed to investigate differences in T cell subset proportions between the patients with untreated early rheumatoid arthritis (ueRA, n=43) and healthy controls (HC, n=14). OPLS-DA score scatter plot showing the separation of ueRA and HC based on the differences T cell subset proportions.

## Slide 3
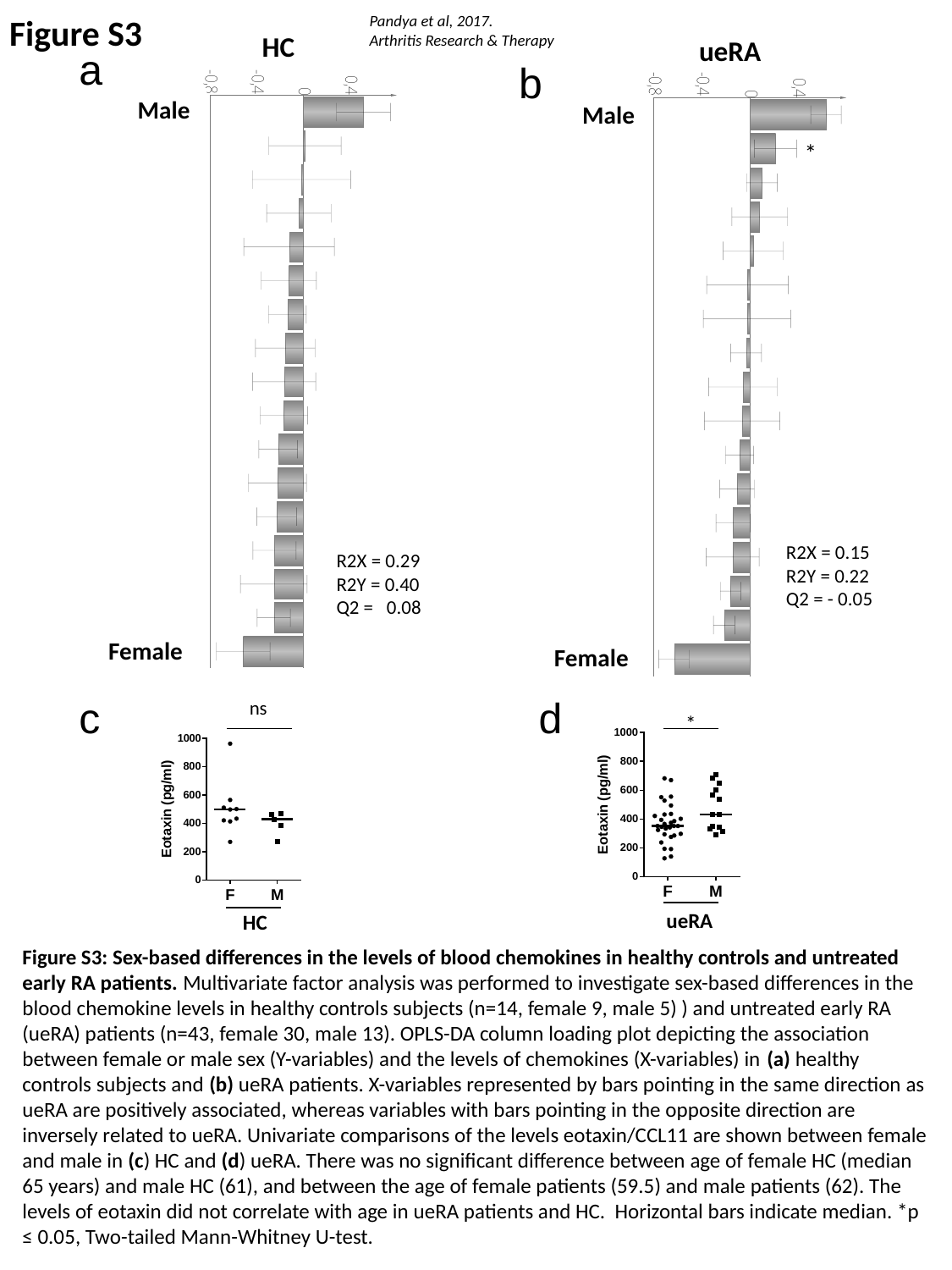

Figure S3
Pandya et al, 2017.
Arthritis Research & Therapy
HC
ueRA
a
b
 Male
 Female
*
 Male
Female
R2X = 0.15
R2Y = 0.22
Q2 = - 0.05
R2X = 0.29
R2Y = 0.40
Q2 = 0.08
c
d
ns
*
ueRA
HC
Figure S3: Sex-based differences in the levels of blood chemokines in healthy controls and untreated early RA patients. Multivariate factor analysis was performed to investigate sex-based differences in the blood chemokine levels in healthy controls subjects (n=14, female 9, male 5) ) and untreated early RA (ueRA) patients (n=43, female 30, male 13). OPLS-DA column loading plot depicting the association between female or male sex (Y-variables) and the levels of chemokines (X-variables) in (a) healthy controls subjects and (b) ueRA patients. X-variables represented by bars pointing in the same direction as ueRA are positively associated, whereas variables with bars pointing in the opposite direction are inversely related to ueRA. Univariate comparisons of the levels eotaxin/CCL11 are shown between female and male in (c) HC and (d) ueRA. There was no significant difference between age of female HC (median 65 years) and male HC (61), and between the age of female patients (59.5) and male patients (62). The levels of eotaxin did not correlate with age in ueRA patients and HC. Horizontal bars indicate median. *p ≤ 0.05, Two-tailed Mann-Whitney U-test.

## Slide 4
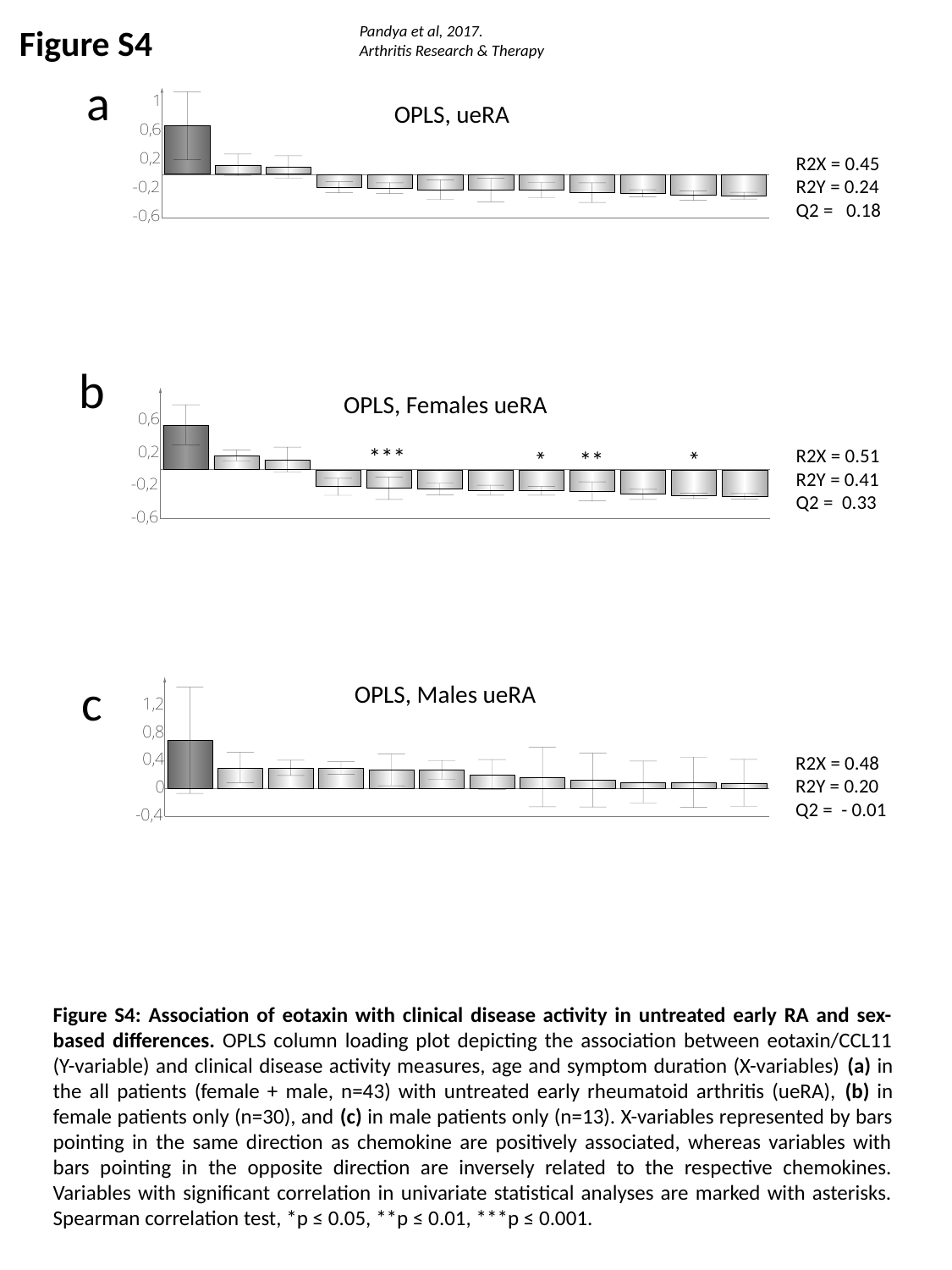

Figure S4
Pandya et al, 2017.
Arthritis Research & Therapy
a
OPLS, ueRA
R2X = 0.45
R2Y = 0.24
Q2 = 0.18
b
OPLS, Females ueRA
*
*
***
R2X = 0.51
R2Y = 0.41
Q2 = 0.33
**
c
OPLS, Males ueRA
R2X = 0.48
R2Y = 0.20
Q2 = - 0.01
Figure S4: Association of eotaxin with clinical disease activity in untreated early RA and sex-based differences. OPLS column loading plot depicting the association between eotaxin/CCL11 (Y-variable) and clinical disease activity measures, age and symptom duration (X-variables) (a) in the all patients (female + male, n=43) with untreated early rheumatoid arthritis (ueRA), (b) in female patients only (n=30), and (c) in male patients only (n=13). X-variables represented by bars pointing in the same direction as chemokine are positively associated, whereas variables with bars pointing in the opposite direction are inversely related to the respective chemokines. Variables with significant correlation in univariate statistical analyses are marked with asterisks. Spearman correlation test, *p ≤ 0.05, **p ≤ 0.01, ***p ≤ 0.001.

## Slide 5
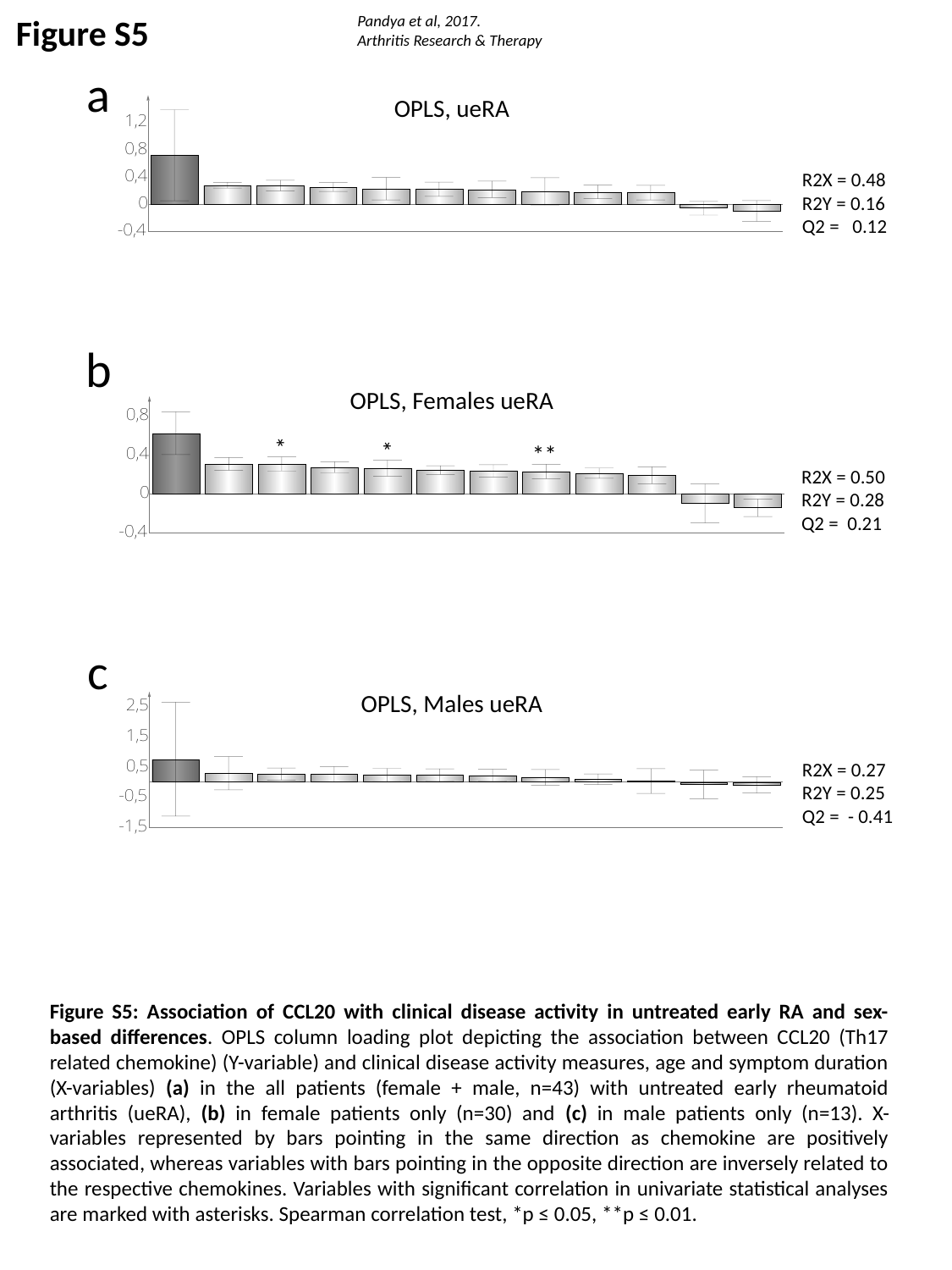

Figure S5
Pandya et al, 2017.
Arthritis Research & Therapy
a
OPLS, ueRA
R2X = 0.48
R2Y = 0.16
Q2 = 0.12
*
*
**
b
OPLS, Females ueRA
R2X = 0.50
R2Y = 0.28
Q2 = 0.21
c
OPLS, Males ueRA
R2X = 0.27
R2Y = 0.25
Q2 = - 0.41
Figure S5: Association of CCL20 with clinical disease activity in untreated early RA and sex-based differences. OPLS column loading plot depicting the association between CCL20 (Th17 related chemokine) (Y-variable) and clinical disease activity measures, age and symptom duration (X-variables) (a) in the all patients (female + male, n=43) with untreated early rheumatoid arthritis (ueRA), (b) in female patients only (n=30) and (c) in male patients only (n=13). X-variables represented by bars pointing in the same direction as chemokine are positively associated, whereas variables with bars pointing in the opposite direction are inversely related to the respective chemokines. Variables with significant correlation in univariate statistical analyses are marked with asterisks. Spearman correlation test, *p ≤ 0.05, **p ≤ 0.01.

## Slide 6
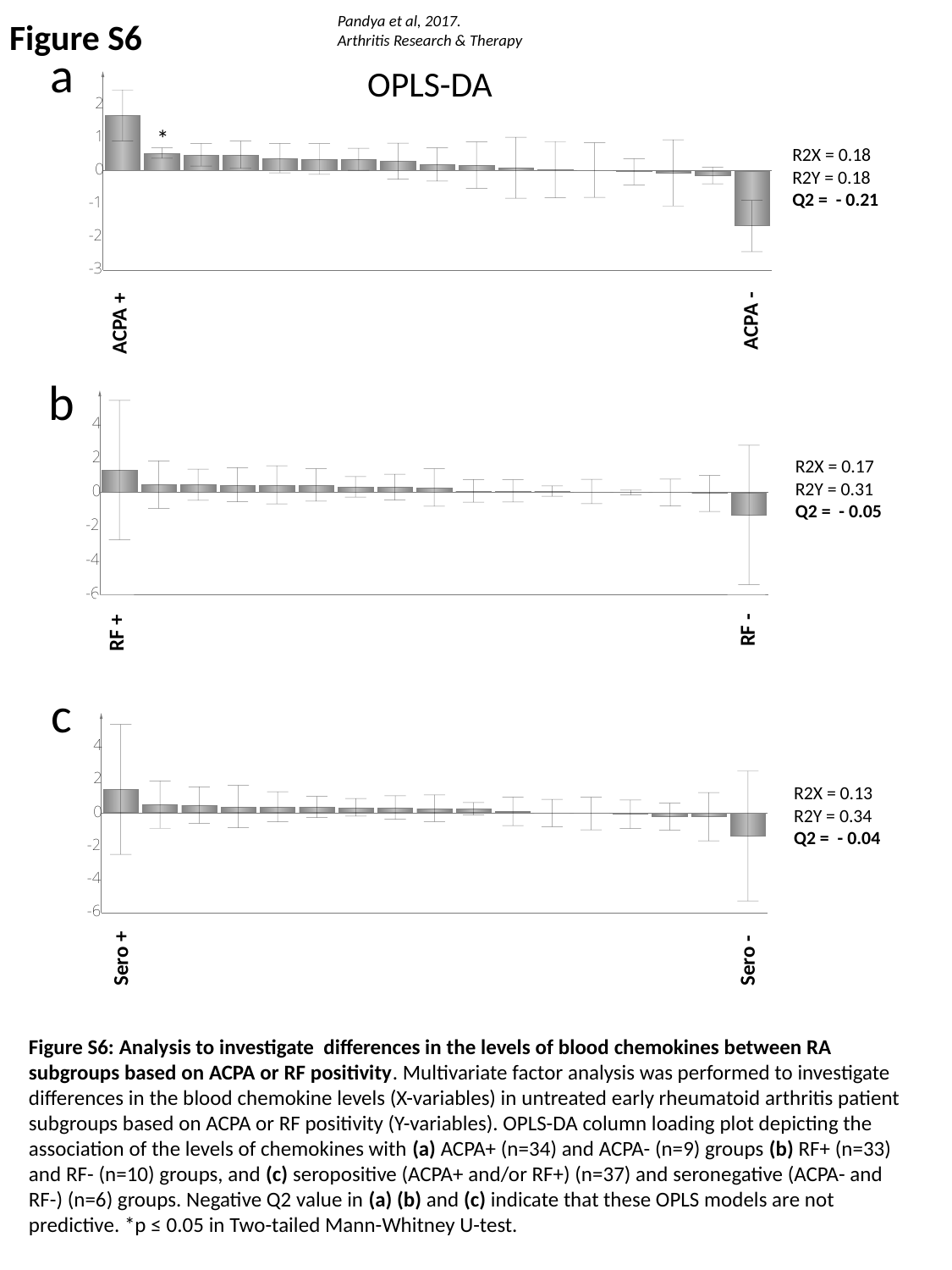

Pandya et al, 2017.
Arthritis Research & Therapy
Figure S6
a
OPLS-DA
*
R2X = 0.18
R2Y = 0.18
Q2 = - 0.21
ACPA +
 ACPA -
b
R2X = 0.17
R2Y = 0.31
Q2 = - 0.05
 RF +
 RF -
c
R2X = 0.13
R2Y = 0.34
Q2 = - 0.04
 Sero +
 Sero -
Figure S6: Analysis to investigate differences in the levels of blood chemokines between RA subgroups based on ACPA or RF positivity. Multivariate factor analysis was performed to investigate differences in the blood chemokine levels (X-variables) in untreated early rheumatoid arthritis patient subgroups based on ACPA or RF positivity (Y-variables). OPLS-DA column loading plot depicting the association of the levels of chemokines with (a) ACPA+ (n=34) and ACPA- (n=9) groups (b) RF+ (n=33) and RF- (n=10) groups, and (c) seropositive (ACPA+ and/or RF+) (n=37) and seronegative (ACPA- and RF-) (n=6) groups. Negative Q2 value in (a) (b) and (c) indicate that these OPLS models are not predictive. *p ≤ 0.05 in Two-tailed Mann-Whitney U-test.
